# Long-Term Characterization of Oxidation Processes in Graphitic Carbon Nitride Photocatalyst Materials via Electron Paramagnetic Resonance Spectroscopy

**DOI:** 10.3390/molecules28186475

**Published:** 2023-09-06

**Authors:** Elizaveta Kobeleva, Ekaterina Shabratova, Adi Azoulay, Rowan W. MacQueen, Neeta Karjule, Menny Shalom, Klaus Lips, Joseph E. McPeak

**Affiliations:** 1 Berlin Joint EPR Laboratory and EPR4Energy, Department Spins in Energy Conversion and Quantum Information Science (ASPIN), Helmholtz-Zentrum Berlin für Materialien und Energie GmbH, Hahn-Meitner-Platz 1, 14109 Berlin, Germany; 2 Department of Chemistry, Ilse Katz Institute for Nanoscale Science and Technology, Ben-Gurion University of the Negev, Beer-Sheva 8410501, Israel; 3 Berlin Joint EPR Laboratory, Fachbereich Physik, Freie Universität Berlin, 14195 Berlin, Germany

**Keywords:** EPR spectroscopy, photocatalysis, carbon nitride, electronic relaxation, semiconductor, oxidation processes, power saturation analysis, light-dependent EPR, electronic structure characterization, graphitic materials

## Abstract

Graphitic carbon nitride (gCN) materials have been shown to efficiently perform light-induced water splitting, carbon dioxide reduction, and environmental remediation in a cost-effective way. However, gCN suffers from rapid charge-carrier recombination, inefficient light absorption, and poor long-term stability which greatly hinders photocatalytic performance. To determine the underlying catalytic mechanisms and overall contributions that will improve performance, the electronic structure of gCN materials has been investigated using electron paramagnetic resonance (EPR) spectroscopy. Through lineshape analysis and relaxation behavior, evidence of two independent spin species were determined to be present in catalytically active gCN materials. These two contributions to the total lineshape respond independently to light exposure such that the previously established catalytically active spin system remains responsive while the newly observed, superimposed EPR signal is not increased during exposure to light. The time dependence of these two peaks present in gCN EPR spectra recorded sequentially in air over several months demonstrates a steady change in the electronic structure of the gCN framework over time. This light-independent, slowly evolving additional spin center is demonstrated to be the result of oxidative processes occurring as a result of exposure to the environment and is confirmed by forced oxidation experiments. This oxidized gCN exhibits lower H_2_ production rates and indicates quenching of the overall gCN catalytic activity over longer reaction times. A general model for the newly generated spin centers is given and strategies for the alleviation of oxidative products within the gCN framework are discussed in the context of improving photocatalytic activity over extended durations as required for future functional photocatalytic device development.

## 1. Introduction

In the context of the global search for new sustainable energy technologies, graphitic carbon nitride (gCN) has gained a lot of attention in the research community as a potentially stable, readily available, and non-toxic photocatalyst for H_2_ production via water splitting and other energy dense molecular syntheses via CO_2_ reduction reactions as well as for environmental remediation via pollutant destruction [1,2]. The success of these proof-of-concept reactions using gCN photocatalysts has prompted research towards the highest possible catalytic output through a variety of surface area enhancements, doping schemes, and other unique applications of graphene-like structural modifications [2] to target the shortcomings of gCN materials. However, the lack of conclusive theoretical explanations or experimental investigations into the understanding of the underlying physical processes restricts gCN photocatalytic efficiency to below industrially applicable values [1]. To date, numerous modifications of gCN structure have been reported to increase catalytic activity without a clear mechanistic reasoning as to how or what process contributes to these increased rates of production. Additionally, gCN continues to suffer from fast quenching of photocatalytic activity and continues to require platinum cocatalysts to improve the stability and reproducibility of catalytic output, preventing gCN from being employed on any large scale as a truly metal-free photocatalyst [3].

**Figure 1:**
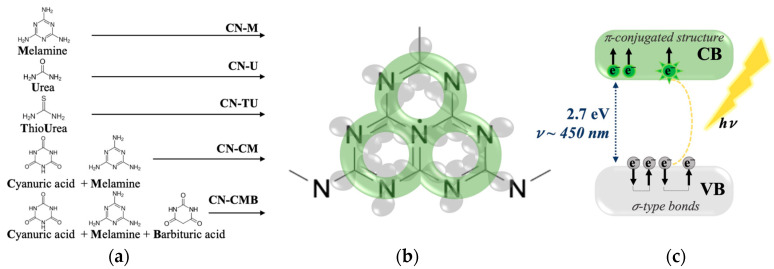
An overview of the structural and electronic properties of gCN. (**a**) Overview of precursors used to synthesize gCN materials; see methods for synthesis procedures. (**b**) The tri-s-triazine unit consists of alternating carbon and nitrogen atoms with bridging nitrogens connecting multiple units into a polymeric structure where single-unit π-conjugation (*green*) and σ-type bonds (*grey*) are shown. (**c**) Schematic illustration of the photoexcitation mechanism of gCN where electrons in the σ-type bonds, which are paired and therefore diamagnetic due to counteracting spin magnetic moments, are excited to the π-conjugated structure where they are then paramagnetic and observable by EPR [4].

Electron paramagnetic resonance (EPR) spectroscopy is particularly well suited to study the electronic structure of gCN materials owing to its non-destructive methods of interrogation and high specificity to paramagnetic electrons. Together with other methods, EPR has been employed to directly monitor spin transitions of photocatalytic species during light-irradiation [2,4]. Figure 1a–c shows an overview of the structural and electronic properties of gCN. The tri-s-triazine units are comprised of alternating carbon and nitrogen atoms, where the electron density of gCN materials allows for electrons in the sp^2^ orbitals of the carbon and nitrogen atoms to form a π-conjugated framework that extends over the entire triazine unit (Figure 1b, *green*). Unpaired electrons distributed over the π-conjugated structure are responsible for photocatalytic activity via transfer from the valence band (σ-type bonds, Figure 1b,c, *grey*) to the conduction band (π-conjugated structure, Figure 1b,c, *green*) under light irradiation [4]. Electrons in the π-conjugated structure are paramagnetic and are therefore observable by EPR spectroscopy. It has been shown that the EPR signal is observable in the dark state, presumably because electrons are already present in the π-conjugated structure; however, under light illumination the EPR double integral intensity increases indicating an increase in the number of spins present [4]. Each triazine unit acts as a contributor to photocatalytic activity and as such, it has been shown in numerous reports that catalytic activity increases with increasing surface area, which could be modified through various synthesis routes [5]. The stability of these materials has previously only been investigated on the scale of hours and a decrease in the efficiency of hydrogen evolution over multiple catalytic cycles has been observed [6]. This timescale is insufficient for commercial applications and therefore the stability should be evaluated over extended durations.

Within this work the gCN electronic structure is further investigated by EPR spectroscopy to describe the current photocatalytic performance of gCN materials and to provide guidelines for further improvement of its photocatalytic efficiency. By providing direct and non-destructive access to the behavior of electrons in materials, examination of the dynamic properties of gCN photocatalysts is made possible in a way that allows increased understanding of the slow alterations to the gCN framework that affect long term stability. This investigation of gCN samples synthesized from various precursors additionally allows for the understanding of correlations between electronic structure and photocatalytic performance with respect to minute changes in synthesis procedures. In this study, we investigate graphitic carbon nitride (gCN) derived from various precursors, namely melamine (CN-M), urea (CN-U), thiourea (CN-TU), as well as supramolecular assemblies of cyanuric acid-melamine (CN-CM) and supramolecular assemblies of cyanuric acid-melamine-barbituric acid (CN-CMB) (Figure 1a). We herein present EPR investigations of gCN materials as a function of time, with saturation and relaxation behavior determined using a combination of both continuous wave and pulse EPR methods to define and probe the environment of the spins and underlying dynamic properties resulting in catalytic activity in gCN materials. Incorporation of laser excitation to classical CW-EPR experiments was similarly employed to study any effects on light response of the samples and to confirm the correlation between light-irradiation and EPR signal as previously reported [4,7]. Interactions with the latent environment were taken into consideration and were further evaluated using forced-oxidation schemes. To evaluate the impact of the herein reported changes in the EPR spectra with respect to time, the catalytic activity via hydrogen production rate measurements were performed using freshly synthesized gCN materials and subsequently reevaluated after twenty months of continuous exposure to normal atmosphere.

## 2. Results

### 2.1. gCN Electronic Stability over Time

The gCN EPR response has been mainly reported to be a single Lorentzian line and associated with unpaired electrons from π-conjugated structures responsible for photocatalytic activity [2,4,5,8]. More recent studies have shown evidence that multiple spins may contribute to the overall CW-EPR spectrum; however, variety in synthesis procedures leading to differences in morphology [5] does not allow for direct comparison of these results [7,9]. In this study EPR spectra were recorded initially and sequentially for 18 months following the initial synthesis of several gCN samples. Significant changes in the EPR spectrum were observed with respect to time and a deviation from the previously reported Lorentzian lineshape was observed in all gCN samples investigated.

**Figure 2:**
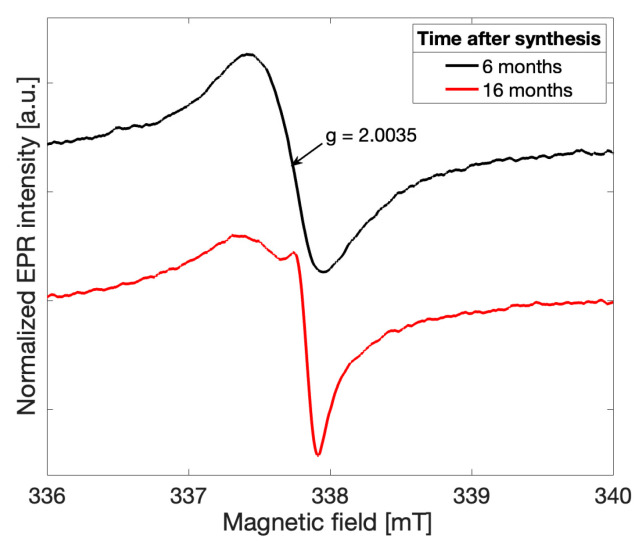
The CW-EPR spectrum recorded after 6 months (*black*) and 16 months (*red*) following successful synthesis of gCN (CN-M) under the same operating conditions. A deviation from a purely Lorentzian lineshape is observed near g = 2.0035 ± 0.00003, as shown by the black arrow in the spectra.

Spectra recorded for all gCN samples investigated exhibited initially a more symmetric lineshape in agreement with previously reported observations [2,4]. As early as six months following the successful synthesis, the overall EPR lineshape was observed to deviate from a single Lorentzian for all five samples in the recorded spectra (Figure 2, *black*). The overall lineshape continued to change over time, providing initial evidence that a change in the electronic structure of the gCN framework has occurred. After 16 months following the successful gCN synthesis, an additional peak may be clearly distinguished (Figure 2, *red*). This observation suggests that an additional paramagnetic species arises in gCN over time and likely alters both the electronic structure of the catalytic material and the physical structure of the bonding character within the triazine units.

**Figure 3:**
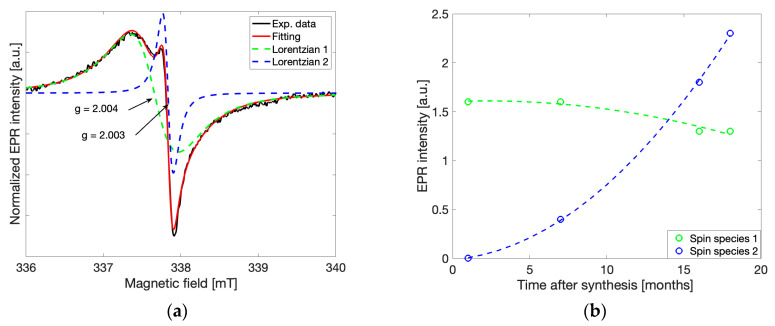
(**a**) The EPR spectrum of gCN (CN-M) shown with individual Lorentzian fits assuming a model comprised of the superposition of two Lorentzian lines, where each independent Lorentzian line is shown at g = 2.004 ± 0.00003 (*green*) and g = 2.003 ± 0.00003 (*blue*). The overall fit is shown as a sum of both individual Lorentzian components (*red*). (**b**) The time-dependent change in the observed EPR peak-to-peak intensities of the two spin species estimated by the fitting procedure is shown in (**a**). The color-scheme corresponding to the individual Lorentzian components is preserved throughout.

A fitting routine which considers two independent spin contributions to the overall spectrum was introduced using a linear combination of two derivative Lorentzian lineshapes according to the following formula,

(1) $$I=m_{1}{\left(\frac{{{\Delta B}_{1}}^{2}}{{\left(B-B_{o_{1}}\right)}^{2}+{{\Delta B}_{1}}^{2}}\right)}^{\prime }+m_{2}{\left(\frac{{{\Delta B}_{2}}^{2}}{{\left(B-B_{o_{2}}\right)}^{2}+{{\Delta B}_{2}}^{2}}\right)}^{\prime }=m_{1}\frac{1}{\pi }\frac{16\left(B-B_{o_{1}}\right){\Delta B}_{1}}{{\left(4{\left(B-B_{o_{1}}\right)}^{2}+{{\Delta B}_{1}}^{2}\right)}^{2}}+m_{2}\frac{1}{\pi }\frac{16\left(B-B_{o_{2}}\right){\Delta B}_{2}}{(4{{\left(B-B_{o_{2}}\right)}^{2}+{{\Delta B}_{2}}^{2})}^{2}}$$

where weighting coefficients $m_{1}$, $m_{2}$, central positions $B_{o_{1}}$, $B_{o_{2}}$ and FWHM (full width at half maximum) ${\Delta B}_{1}$, ${\Delta B}_{2}$ values were allowed to vary within a least squares regression (performed in Matlab, *Mathworks*) [10]. In Figure 3a, the gCN spectrum shown previously in Figure 2 (*red*) is modelled using the above relationship. The overall fit is the sum of two Lorentzian lineshapes such that each independent Lorentzian lineshape can be built using one set of coefficients $[m_{1}$, $B_{o_{1}}$, ${\Delta B}_{1}$] or $[m_{2}$, $B_{o_{2}}$, ${\Delta B}_{2}$] obtained from the fit. The same fitting routine and initial parameters were utilized for all five investigated samples. Spectra recorded at different observation times and different microwave powers were found to be in good agreement throughout all samples. While the observed g-values of the two peaks were constant, the intensities and linewidths varied with microwave power, time, and temperature.

Here, the wider lineshape (Figure 3a, *green*, ΔB_pp_ = 0.6 mT) labeled as spin species 1, corresponds to unpaired electrons distributed over the π-conjugated structure of the triazine unit of gCN, also known to be responsible for photocatalytic activity, as both linewidth and g-value agree well with previous reports [2,4]. The origin of the narrow lineshape (Figure 3a, *blue*, ΔB_pp_ = 0.1 mT), labeled spin species 2, has not been investigated before.

By separating the two Lorentzian contributions to the overall EPR spectrum via the fitting routine described above, the peak-to-peak intensities of each species were determined for spectra recorded under the same conditions at different time points between 2 and 18 months after synthesis (Figure 3b). The intensity of the line associated with spin species 2 (Figure 3b, *blue*) increases drastically over time, while the intensity of the line associated with spin species 1, typically attributed to catalytically active spins, (Figure 3b, *green*) decreases slowly over the same time duration. The exponential increase in the intensity at g = 2.003 ± 0.00003 and slow decrease in the intensity at g = 2.004 ± 0.00003 provides insight into the dynamic nature of gCN. It is likely that the second spin species (g = 2.003) forms spontaneously from interactions with the environment, followed by a decreasing concentration of the spin species associated with photocatalytic activity (g = 2.004). Such behavior provides initial implications that a degradation process occurs over time.

In newly synthesized gCN materials, the EPR spectral component corresponding to spin species 2 is negligible, so the gCN spectrum in principle may be described with a single Lorentzian lineshape and has been shown by previous groups where a single symmetric Lorentzian lineshape in the gCN EPR spectrum was observed [2,4,5,8]. Deviation from a Lorentzian shape as presented in this work is only observable following significantly long times after synthesis. Nevertheless, evidence of the presence of the second spin species long after the synthesis has made it possible to reconstruct the contributions of both spin species in recently synthesized samples allowing for investigation of the potential effects on photocatalytic activity on a relevant timescale and are herein reported.

### 2.2. Saturation and Relaxation Behavior

To investigate the independent behavior of the two observed Lorentzian contributions to the gCN EPR spectrum, power saturation behavior was recorded and the fitting procedure described previously was applied to each EPR spectrum recorded at varying microwave power levels. Power saturation curves interrogate the dependence of EPR amplitude on the applied B_1_ field such that saturation behavior may be used for the determination of electronic longitudinal (T_1_ or spin-lattice) and transverse (T_2_ or spin-spin) relaxation rates of the system because these processes are closely correlated allowing for their estimation from CW-EPR experiments [10,11].

**Figure 4:**
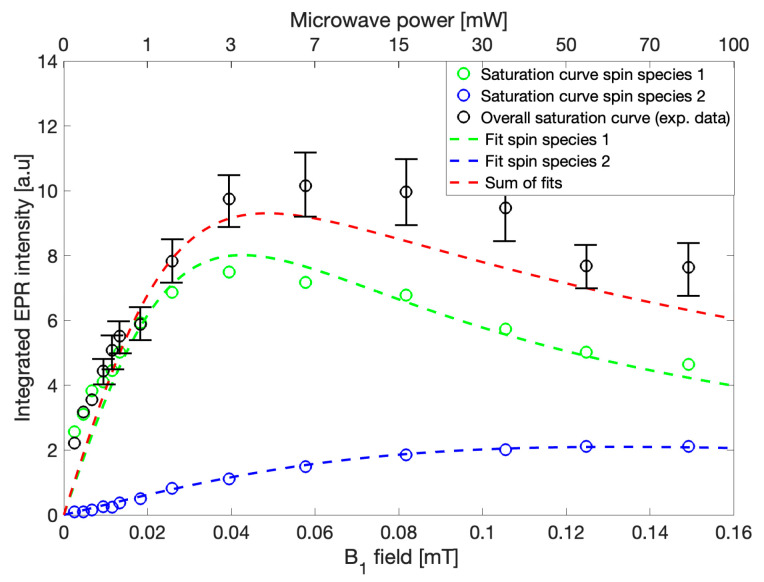
Simulated power saturation curves recorded via CW-EPR for the independent spin species identified in Figure 3a shown with the color scheme preserved (g = 2.004, *green*, g = 2.003, *blue*) with fits to the simulated data shown (*green and blue dashed lines*) assuming homogeneous saturation behavior. The experimentally recorded power saturation data are shown (*black open circles*) with the sum of the two simulated power saturation curves overlaid *(red dashed line*).

EPR spectra were recorded sequentially using microwave powers from 0.1 mW to 100 mW while keeping all other parameters constant. The maximum integrated intensities of the recorded spectra were plotted against B_1_, which is proportional to the square root of microwave power (Figure 4, *black circles*). Because the spectra within this power range may be fit using two Lorentzian lines, the individual intensities of each of the two lines were constructed via the previously described fitting routine and were similarly plotted against B_1_ (Figure 4, g = 2.004, *green circles*, and g = 2.003, *blue circles*), to yield simulated power saturation curves. Fits to the power saturation data were performed using the analytical formula for homogeneous saturation as follows,

(2) $$I=\frac{B_{1}I_{m}^{0}}{1+B_{1}^{2}\gamma^{2}T_{1}T_{2}}$$

where I represents the integrated intensity of the EPR signal, B_1_ is the magnetic field of the microwaves, $I_{m}^{0}=\underset{B_{1}\to 0}{\lim}\left(\frac{I}{B_{1}}\right)$, γ is the gyromagnetic ratio, T_1_ is the spin-lattice relaxation time, and T_2_ is the spin-spin relaxation time [11]. This model provides an estimation of T_1_ relaxation times under the assumption that T_2_ may be determined by the linewidth of a purely Lorentzian signal and no additional unresolved contributions to relaxation are present [11]. Both simulated saturation curves constructed based on individual signal intensities were found to be adequately described independently using a homogeneous saturation model (Figure 4, *green and blue dashed lines*) allowing for the conclusion that the gCN EPR spectrum consists of two spin centers at approximately g = 2.004 and g = 2.003. Since the involved spin species are not identical, the overall saturation of the system cannot be described by a homogeneous saturation model. Due to the complexity of non-homogeneous saturation and the many possible processes involved, an analytical description for the general case is not valid; however, the saturation behavior of gCN resulting from the recorded EPR spectra may be described as the sum of two separate power saturation curves within reasonable error (Figure 4, *red dashed line*). The significantly higher experimental values when compared to those obtained from the sum of the independently derived saturation curves could be explained by consideration of the integrated noise contributions to the overall intensity, which is not considered in the fitting routine [12]. Such discrepancies between the observed and calculated intensities may also arise from the assumption of absolute independence with respect to the simulations of the two spin systems, which is unlikely given their close proximity to one another. Therefore, additional weakly coupled interactions resulting in cross-correlations between the spin centers might occur and affect the saturation and relaxation behavior of each individual spin system [13,14]. Nevertheless, relaxation behavior may be qualitatively determined from these experiments such that spin species 1 (g = 2.004) which is represented by a wider EPR linewidth and saturation at lower microwave powers would typically correspond to faster T_2_ and slower T_1_, respectively, in comparison to spin species 2 (g = 2.003), which is instead described by a narrower EPR linewidth and later saturation, which would conversely have slower T_2_ and faster T_1_, respectively. This routine provides only an estimation and is not a direct observation; therefore, these results were further evaluated using complimentary pulse EPR and continuous wave saturation recovery EPR experiments.

Following qualitative analysis of relaxation via CW experiments, relaxation time constants of gCN materials were measured directly by CW saturation recovery EPR techniques at X-band and by pulse EPR techniques at Q-band. CW saturation recovery measurements were preferentially performed at X-band due to the presumably very short T_2_ time constants which therefore render pulse techniques at this frequency ineffective due to low echo intensities after consideration of the dead time of the instrument. The T_1_ times estimated from the constructed saturation curves agree qualitatively with those obtained via continuous wave saturation recovery experiments. The relaxation times observed in gCN materials are not reported in the literature; however, these values may be compared with those observed in similar materials, for example, N-doped graphene oxide is reported to have similarly long T_1_ relaxation [15,16,17]. Because only T_1_ relaxation times increase with microwave frequency and T_2_ is primarily frequency-independent [18,19], the two-pulse Hahn-echo sequence for measuring T_2_ relaxation at Q-band was used for T_2_ relaxation measurements. The resulting echo lasted sufficiently beyond the dead time of the instrument to enable not only T_2_ measurements but also the three-pulse inversion recovery sequence for measuring T_1_ relaxation at Q-band. In all experiments for all gCN compounds, similar and consistent results were obtained. For all relaxation data, fits using a biexponential function resulted in lower standard deviation in a residual analysis (Figures S1–S3 from Supplementary Materials). Mono-exponential functions were considered, but the residual analysis demonstrated nonlinear deviation above the noise levels in both cases. The spin–lattice (T_1_) relaxation times and spin–spin (T_2_) relaxation times varied by factors of only about two to three between all samples investigated and may be explained by differences in the morphologies of the samples due to diverse synthesis pathways [5], different spin concentrations influencing dipole coupling, and varying magnitudes of contributions from the first and second species and the resulting effects of cross correlated relaxation [13,14]. Further descriptions of the relaxation data and the various fitting approaches may be found in the supporting information. The multi-component relaxation behavior observed via CW saturation recovery at X-band and via pulse experiments at Q-band provide further support that two spin species are present in the gCN systems investigated.

A summary of relaxation times observed is given in Table 1. Longer T_1_ values, which correspond to a lower degree of spin-lattice interactions, together with shorter T_2_ values, which correspond to a greater contribution from spin-spin interactions, may indicate high delocalization and give some idea of the mobile character of spin species 1 (g = 2.004). These spins are primarily localized to the π-conjugated structure of the triazine rings and are responsible for photocatalytic activity [4]. On the contrary, shorter T_1_ values and longer T_2_ values observed for spin species 2 (g = 2.003) indicate a much greater interaction with the lattice or surrounding environment and a lower contribution from spin–spin interactions which may instead be correlated with an immobilized spin system and suggests that this species may not directly participate in the photocatalytic activity of the material.

### 2.3. Light Response

**Figure 5:**
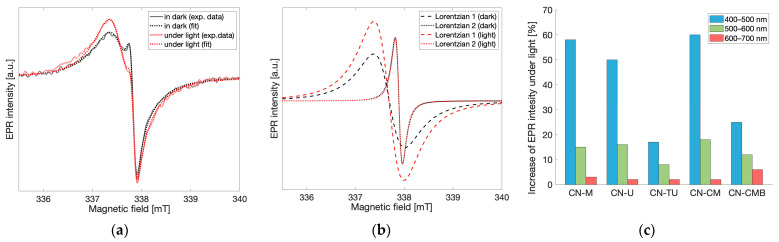
(**a**) EPR spectra of gCN (CN-M) recorded under dark conditions and under light irradiation (λ = 500–600 nm). (**b**) Individual Lorentzian lines obtained from the fits of the light irradiated and dark state spectra (**c**) The relative change of the EPR peak-to-peak intensity of spin species 1 (g = 2.004) under light irradiation using varying wavelengths of light compared to the EPR intensity recorded in the dark state for different gCN samples.

To understand the EPR signal response to light and therefore define the probabilities of each spin species exerting any effects on gCN photocatalytic performance, light-dependent EPR measurements were performed at X-band. For all gCN materials investigated, an increase in the resulting EPR signal was observed in response to irradiation by visible light. The EPR spectra recorded under dark conditions and under continuous light irradiation for the melamine-based gCN material are shown in Figure 5a. After applying the fitting routine described previously to differentiate the contributions from each of the spin species observed (Figure 5b), the EPR spectrum under light irradiation shows that the low field, wider component corresponding to spin species 1 (g = 2.004) increases by 60% when under irradiation, while the high field, narrower component corresponding to spin species 2 (g = 2.003) remains unchanged. Similar light-response behavior was observed for all investigated gCN materials and wavelengths of light in the range 400–700 nm. This demonstrates that only spin species 1 is light-active while species 2 which appears months after synthesis is light-inactive. Prolonged irradiation using white light resulted in further increase in the EPR peak-to-peak intensity of spin species 1 while the EPR intensity of spin species 2 remained unchanged. Similarly, the reverse effect was observed when removing the sample from light. After keeping the gCN materials in the dark for prolonged times, decreases in the EPR intensity of spin species 1 were observed while the EPR intensity of spin species 2 remained unchanged. These findings bring forth the implication that spin species 1 is likely associated with the spins responsible for photocatalytic activity [2,3,4]. The light-inactive character of spin species 2 instead implies that these spins likely do not contribute positively to photocatalysis. Rather, in conjunction with the time-dependent increase in the EPR intensity of spin species 2 following synthesis of the material, it is likely that this species may be indicative of the formation of degradation products within the gCN material. In much later stages of this study, a third spin species was detected in some but not all of the gCN samples investigated; however, this contribution to the overall signal was significantly lower than the two signals reported and was not well reproduced such that it was not considered for the further analysis (Figure S4 from Supplementary Materials). This signal likely corresponds to further degradation processes and may be attributed to an additional localized spin species as it does not respond to light; however, a thorough analysis of this contribution was beyond the scope of this work.

To better investigate the light-dependence of the EPR signals in the spectra obtained for gCN, EPR measurements were performed for all gCN samples while exposed to continuous light irradiation using the following 100 nm bands: 400–500 nm, 500–600 nm, 600–700 nm and in the dark state as a reference. Each recorded spectrum was fit using two Lorentzian lines, as described previously, and the relative change of the EPR peak-to-peak intensities between the dark state and under illumination at each of the irradiation bands for both Lorentzian lines was calculated independently. The relative changes in the EPR intensity at different bandwidths of excitation were normalized to the same photon flux while the linear dependence of the light-induced increase in EPR intensity with respect to photon flux was verified separately using a halogen cold light source (Schott KL 2500 LCD and Thurlby Thandar TSX1820P). As stated previously, the intensity of spin species 2 remained unchanged in all irradiation conditions. Therefore, only the relative change in the EPR intensity of spin species 1 is shown in Figure 5c. For all gCN materials investigated, the maximum increase in EPR intensity was observed when the spectrum was recorded using light irradiation bandwidths of 400–500 nm, while the increase in EPR intensity was significantly lower for the 500–600 nm bandwidth and even smaller for the 600–700 nm bandwidth. Noticeably, this behavior correlates very well with UV-Vis spectra previously recorded for gCN materials [20,21]. Though the EPR spectrum of gCN materials is typically interpreted as a single component, separating gCN EPR spectra into multiple components and considering only the light-active species in the assessment of light-dependent properties leads to better predictions of photocatalytic activity [4,7]. While this experiment allows for the comparison of the light response within the material under irradiation from different wavelengths, comparability between the overall activity of different gCN materials with respect to surface effects must be considered. It has been shown that photocatalytic activity mainly occurs on the surface of the material [22,23,24]. However, due to morphological differences within the materials impacting the diffraction of the incident light, as well as variations in porosity and therefore variations in both density and surface area, the number of spins exposed to irradiation may vary greatly between different gCN materials. Therefore, a comparison between materials according to the light-induced EPR response was not considered due to the difference in the responses between samples (Figure 5c) which primarily corresponds to the difference in numbers of spins accessible by light irradiation and not directly the total number of photoactive spins within the material.

### 2.4. Oxidation Effects

In order to investigate the origin of spin species 2 (g = 2.003) which was thought to result from interactions with the environment leading to the observed time-dependent changes in the EPR spectra, attempts were made to elicit similar processes artificially while recording the EPR spectrum both before and after the elicited process. The gCN materials were stored in non-sealed vials open to the air, where both reactive oxygen and water are present [25]. Since gCN is a photocatalyst, it is possible that H_2_ may be formed via water splitting reactions using water from the air even without special conditions. To determine if this is the case, the relative humidity was increased in the sample tube and any resulting effects on the observed EPR lineshape were recorded. This was performed in two ways: by blowing water vapor into the sample tube and by adding water dropwise directly to the sample tube. In neither of these experiments were any changes to the lineshape observed.

**Figure 6:**
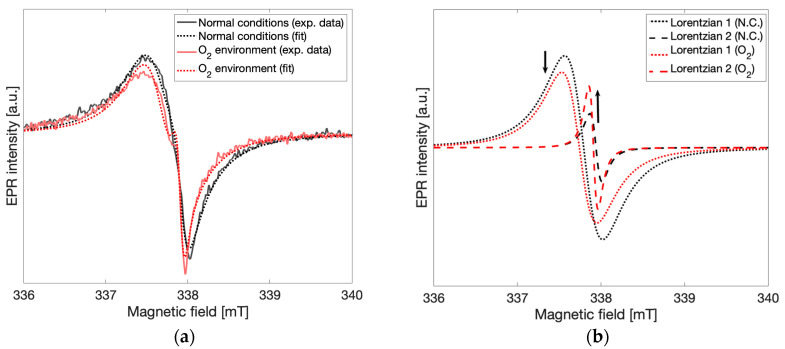
(**a**) The change in the EPR spectrum observed under normal atmospheric conditions and after exposure to an oxygen enriched environment. (**b**) The individual Lorentzian lines obtained from the fitting routine correspond to spin species 1 (g = 2.004) and spin species 2 (g = 2.003) for both atmospheric and oxygen enriched conditions, shown on the same scale as the experimental data.

To test the effects of oxygen in the environment on the spectral lineshape, the sample tube with gCN powder was exposed to an oxygen-enriched environment by replacing the atmosphere of the EPR sample tube with oxygen. EPR spectra were recorded immediately after oxygen was introduced and continuously for 8 h. In Figure 6a spectra taken in the oxygen-enriched environment and in normal atmospheric conditions are shown for comparison. Both an increase in the signal corresponding to spin species 2 and a decrease in the signal corresponding to spin species 1 were observed. In Figure 6b Lorentzian lines calculated from the fitting routine described above and corresponding to spin species 1 (g = 2.004, *dotted lines*) and spin species 2 (g = 2.003, *dashed lines*) are shown under normal atmospheric conditions (*black*) and oxygen-enriched conditions (*red*). The observed relative change in the peak-to-peak intensities of the Lorentzian lines demonstrates that concentrations of spin species 2 increased while concentrations of spin species 1 decreased after oxygen enrichment in a manner similar to those observed to be taking place slowly over time. Direct oxidation of the gCN material was attempted via treatment with aqueous H_2_O_2_ solution (3%) and the EPR response before and after treatment was recorded. The obtained results demonstrated a similar effect where the signal from spin species 2 increases in intensity while the signal from spin species 1 decreases. This effect is quickly followed by additional degradation of the material, characterized by numerous additional overlapping signals (Figure S5 from Supplementary Materials) [9]. Further analysis of these results was not pursued due to low SNR from the increased microwave absorption of aqueous solutions. From these experiments taken together, it may be concluded that a local increase in oxygen concentration interacting with the gCN material leads to similar spectral changes to those observed in the time-dependent response, providing unambiguous support for oxidation processes as the primary influence on the time-dependent appearance of spin species 2 and may be attributed to new spin centers formed via the interaction of gCN with atmospheric oxygen.

### 2.5. Impact on Catalytic Performance

**Figure 7:**
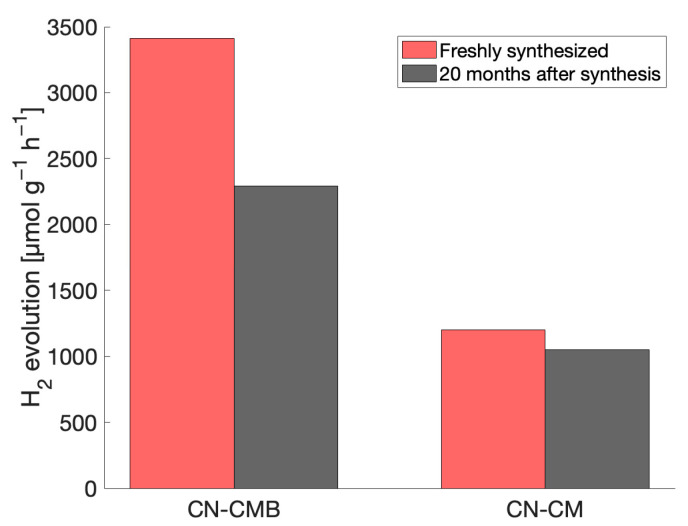
Hydrogen evolution rates observed for two different gCN materials, CN-CM and CN-CMB, when measured immediately after synthesis and 20 months after synthesis.

To determine the effects from the time-dependent structural changes in gCN materials observed by EPR on their photocatalytic performance, hydrogen evolution measurements on fresh gCN materials and gCN materials synthesized 20 months prior were conducted. Among the gCN materials studied, CN-CM and CN-CMB were chosen on account of their notably high reproducibility and stability in terms of hydrogen evolution rates [21]. As shown in Figure 7, both CN-CM and CN-CMB materials demonstrated a decrease in the observed H_2_ evolution rate over time, with the appearance of spin species 2 in the EPR spectrum. The introduction of spin species 2 is accompanied by a reduction in spin species 1 which is presumed to be the spin species predominantly responsible for photocatalytic activity and correlates well with a decrease in the overall concentration of photoactive electrons over time, negatively impacting the photocatalytic H_2_ evolution rate. The oxidative processes in the gCN materials are detrimental for the photocatalytic performance of both CN-CM and CN-CMB and is representative of all gCN materials investigated.

## 3. Discussion

**Figure 8:**
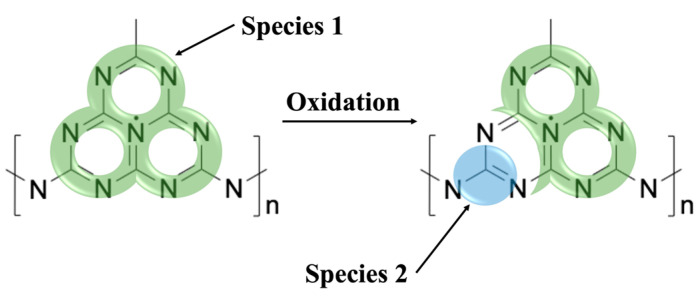
Potential oxidative processes in the gCN structure where delocalization of spin species 1 (g = 2.004) is highlighted in green while the introduction of spin species 2 (g = 2.003) associated with radicals formed via oxidation is highlighted in blue.

In Figure 8, a structural model is given depicting the potential sites for spin centers that would likely result in the EPR signal (spin species 2, g = 2.003) observed to appear and increase over time in gCN materials. While spin species 1 (g = 2.004) is attributed to electrons delocalized over the entire π-conjugated structure of the triazine unit, it is now suggested that the signal associated with spin species 2 originates from oxidation-produced radicals localized at carbon atoms [26]. These radicals may be formed by either the introduction of an oxygen-centered species (denoted with R in Figure 8) or via oxidation of carbon atoms directly, most likely at the border of the triazine unit, without incorporation of the reactive oxygen into the molecule [17,25,27]. Alternatively, reactive oxygen could attack the nitrogen atoms to form nitrogen-centered radicals; however, the g-value of the narrow line (spin species 2, g = 2.003) is in much better agreement with the formation of carbon-centered radicals [28]. Formation of radicals via oxidative damage destroys the local electronic configuration in a way that decreases the total area of delocalization within the π-conjugated structure accessible by the electrons [6,25]. Thus, with increasing concentration of radicals formed from oxidative damage in gCN materials, the concentration of photoactive delocalized electrons decreases simultaneously. This results in quenching of the overall photocatalytic activity.

The oxidation mechanism proposed aligns well with the herein reported observations based on the experiments performed. EPR spectra recorded from CW experiments reveal simultaneous contributions from multiple spin centers as early as six months after synthesis of gCN materials, where two spin species, one of which is highly mobile and likely responsible for photocatalytic activity as shown by the observed relaxation behavior, light-response and literature support [2,4,7,8], while another demonstrates both relaxation behavior and a lack of any light-response typical of immobilized states or isolated radicals [17,27]. Both processes, the increasing concentration of spin species 2 (g = 2.003) and the decreasing concentration of spin species 1 (g = 2.004) were observed to occur with time. Evidence of the influence of reactive oxygen species, and not water present in the atmosphere, on the EPR lineshape and thus the spin centers present is provided by the EPR measurements performed both before and after exposure to water, oxygen-enriched environments, and H_2_O_2_ solutions. The detrimental behavior of these interactions is demonstrated by the reduced photocatalytic H_2_ production rates in the newly synthesized and oxidized gCN materials investigated.

These findings concerning the long-term stability of gCN materials indicate a need for further investigation of the degradation processes and how they might be alleviated in large-scale applications. As has been demonstrated by the observed reductions in photocatalytic performance, possibilities to prevent oxidative damage are necessary enroute to functional devices. Methods of oxidation may be reduced by close attention to handling of the materials during synthesis and while in use such that the H_2_ production rate is maintained over long durations.

## 4. Materials and Methods

### 4.1. Synthesis

The gCN samples investigated in the present work were synthesized from different precursors (Figure 1a) with slightly different synthesis procedures.

#### 4.1.1. Synthesis of Supramolecular Assemblies

Cyanuric acid-melamine (CM) complex was prepared by mixing cyanuric acid (0.51 g, 4 mmol) and melamine (0.50 g, 4 mmol) in a 1:1 molar ratio in 40 mL DI water for 12 h in an automatic shaker (KS-260, IKA-Werke GmbH & Co. KG, Staufen, Germany). The obtained solid precursor was filtered and then dried at 60 °C in a vacuum oven. Cyanuric acid-melamine-barbituric acid (CMB) complex was synthesized by mixing in a 1:1:0.05 molar ratio, following the same procedure as for the CM complex.

#### 4.1.2. Preparation of CN Materials

The gCN materials were prepared via thermal condensation of each precursor; melamine (M), urea (U), thiourea (TU), cyanuric acid and melamine following supramolecular preorganization methods (CM), and cyanuric acid, barbituric acid, and melamine following supramolecular preorganization methods (CMB) at 550 °C for 4 h under an inert N_2_ atmosphere in a muffle furnace (heating rate of 2.5 °C min^–1^ from room temperature). All gCN products were collected and labelled as CN-X, where X represents the corresponding precursor.

### 4.2. Electron Paramagnetic Resonance (EPR) Measurements

Powder gCN samples were placed in quartz/suprasil 4 mm O.D. EPR tubes. Since the density and thus the spin concentrations vary considerably between different gCN powders, from 10^12^ spins per milligram for CN-M to 10^14^ spins per mg for CN-CMB, the volume of each sample was carefully controlled rather than the mass. Each tube was filled with approximately 0.25 cm^3^ powder, which is equal to 2 cm sample height in the EPR tube and is equivalent to the active space of the resonant cavity. EPR measurements were performed using a Magnetech MS-5000 benchtop X-band spectrometer with an operating frequency of 9.45 GHz at room temperature and ambient light access. Modulation amplitude and frequency were 0.1 mT and 100 kHz. CW spectra were recorded using a microwave power of 15 mW, while for power saturation measurements, microwave power was varied from 0.05 mW to 100 mW. Comparative CW spectra were normalized to the integrated intensity of the absorption signal, when appropriate. The standard deviation and total variance of the g-values reported were calculated from three consecutive CW measurements using N@C_60_ as a reference standard and were found to be ±0.00003 and ±0.00006, respectively.

Pulse EPR measurement were performed using an Elexsys E580 spectrometer (Bruker Biospin, Ettlingin, Germany) with a Q-band resonator (33.75 GHz) and microwave power of 20 mW. Samples were placed in quartz/suprasil 1.6 mm EPR tubes. Saturation recovery measurements were performed using a modified Bruker E500T spectrometer described previously [29].

For light-dependent measurements a Magnettech MS-5000 EPR spectrometer and a supercontinuum laser, a SuperK Fianium equipped with a Varia filter (NKT Photonics, Birkerød, Denmark), were used. The pulse repetition rate of the laser was 78.2 MHz, much faster than the rate of the CW EPR measurements. The EPR spectrometer and laser were connected in such a manner that the laser beam was directed into the middle of the resonator and uniformly illuminated one face of the sample volume, while outside of the resonator dark conditions were maintained.

EPR spectra were recorded for each gCN material investigated. CW spectra presented herein are shown for CN-M, but the spectral descriptions and resulting observations and conclusions are relevant for all gCN materials investigated unless otherwise stated.

### 4.3. Photocatalytic H_2_ Production Measurements

The hydrogen evolution reaction was conducted under a constant temperature of 25 °C in a sealed quartz vessel held by a jacketed beaker thermally regulated with a circulating cooling system. In each, 15 mg of CN material was suspended via sonication for 15 min in an aqueous solution containing 17.1 mL of DI water, 1.9 mL of TEOA as the hole scavenger, and 19.6 µL of H_2_PtCl_6_ solution (3%wt Pt relative to CN-X photocatalyst mass). After purging with Ar to remove residual air in the vessel, Pt cocatalyst nanoparticles were in-situ photodeposited on the CN-X catalyst surface under white light-emitting diode (LED) light irradiation (BXRA-50C5300, 100 W, λ > 410 nm, Bridgelux, Fremont, CA, USA) for 0.5 h with continuous stirring (600 rpm). Gas chromatography (Agilent 7820 GC) was employed to monitor the amount of evolved H_2_ gas in the headspace every 0.5 h.

## Figures and Tables

**Table 1:** Relaxation time constants observed in continuous wave saturation recovery EPR experiments at X-band, two-pulse echo decay EPR experiments at Q-band, and three-pulse inversion recovery EPR measurements at Q-band after fitting with a biexponential model.

	T_1_ [μs] X-Band CW Saturation Recovery		T_2_ [ns] Q-Band Hahn-Echo		T_1_ [μs] Q-Band Inversion Recovery	
	1st Species	2nd Species	1st Species	2nd Species	1st Species	2nd Species
CN-M	40 ± 10	4 ± 1	590 ± 30	3900 ± 200	450 ± 90	90 ± 20
CN-U	70 ± 10	4 ± 1	1300 ± 300	4800 ± 1200	370 ± 70	80 ± 10
CN-TU	-	-	700 ± 100	2600 ± 400	410 ± 60	100 ± 20
CN-CM	100 ± 20	15 ± 3	500 ± 100	1700 ± 400	700 ± 100	130 ± 20
CN-CMB	50 ± 5	2.0 ± 0.2	700 ± 50	1570 ± 80	520 ± 30	85 ± 5

## Data Availability

Data will be made available upon reasonable request.

## Supplementary Materials

The following supporting information can be downloaded at: https://www.mdpi.com/article/10.3390/molecules28186475/s1, Figure S1: T_2_ relaxation decay curve measured via the Hahn-echo sequence in Q band and fit with a biexponential function for CN-CMB. Figure S2: T_1_ relaxation recovery curve measured via the 3-pulse inversion recovery sequence in Q band and fit with a biexponential function for CN-CMB. Figure S3: Example of residual analysis after subtraction of fits via either exponential or biexponential functions for T_1_ relaxation data. Figure S4: EPR spectrum of gCN fit with a superposition of 3 Lorentzian lines. Figure S5: EPR spectrum of gCN after adding H_2_O_2_.
