# Supplementary material for: Long-Term Characterization of Oxidation Processes in Graphitic Carbon Nitride Photocatalyst Materials via Electron Paramagnetic Resonance Spectroscopy
Source: Molecules. 2023 Sep 6;28(18):6475. doi: 10.3390/molecules28186475 (PMC10537412; doi:10.3390/molecules28186475)
Supplement: Supplementary file 1 [file molecules-28-06475-s001.zip › molecules-2557272-supplementary.pdf]

Supplementary Materials

List of Figures

Figure S1. T_2 relaxation decay curve measured via the Hahn-echo sequence in Q band and fit with a biexponential function for CN-CMB.

Figure S2. T_1 relaxation recovery curve measured via the 3-pulse inversion recovery sequence in Q band and fit with a biexponential function for CN-CMB.

Figure S3. Example of residual analysis after subtraction of fits via either exponential or biexponential functions for T_1 relaxation data.

Figure S4. EPR spectrum of gCN fit with a superposition of 3 Lorentzian lines. The g-values are as follows: Lorentzian 1, 2.004, Lorentzian 2, 2.003, Lorentzian 3, 2.008.

Figure S5. EPR spectrum of gCN after adding H_2O_2, 3%, aqueous, to the sample tube and subsequently drying with nitrogen gas.

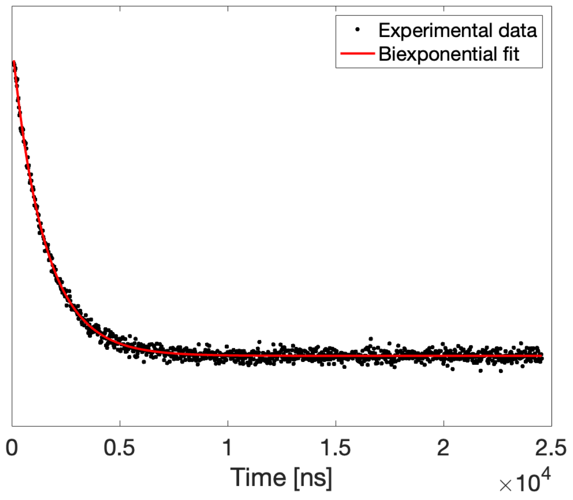

Figure S1: T_2 relaxation decay curve measured via the Hahn-echo sequence in Q band and fit with a biexponential function for CN-CMB.

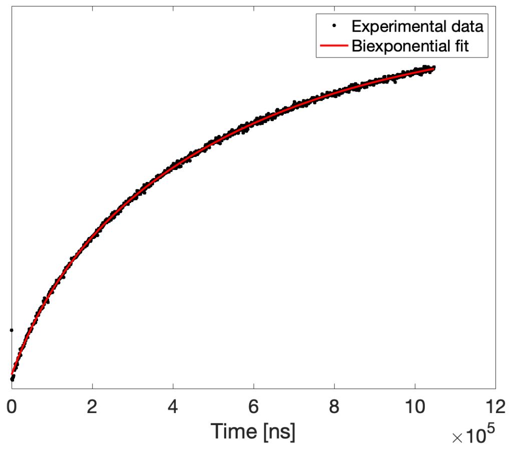

Figure S2: T_1 relaxation recovery curve measured via the 3-pulse inversion recovery sequence in Q band and fit with a biexponential function for CN-CMB.

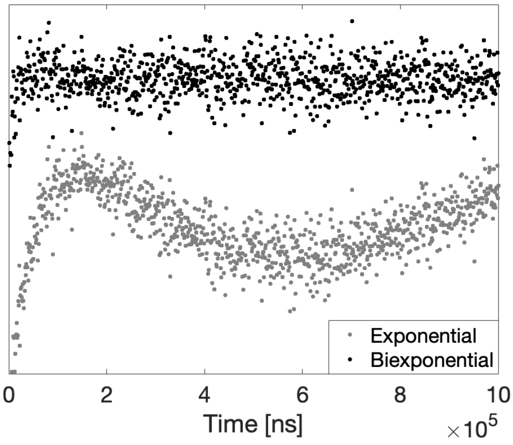

Figure S3: Example of residual analysis after subtraction of fits via either exponential or biexponential functions for T_1 relaxation data.

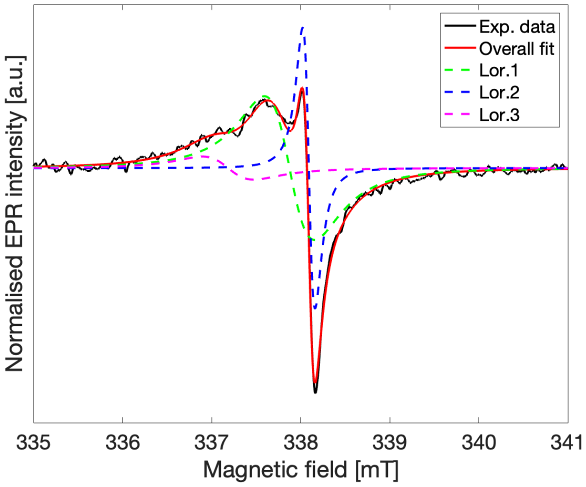

Figure S4: EPR spectrum of gCN fit with a superposition of 3 Lorentzian lines. The g-values are as follows: Lorentzian 1, 2.004, Lorentzian 2, 2.003, Lorentzian 3, 2.008.

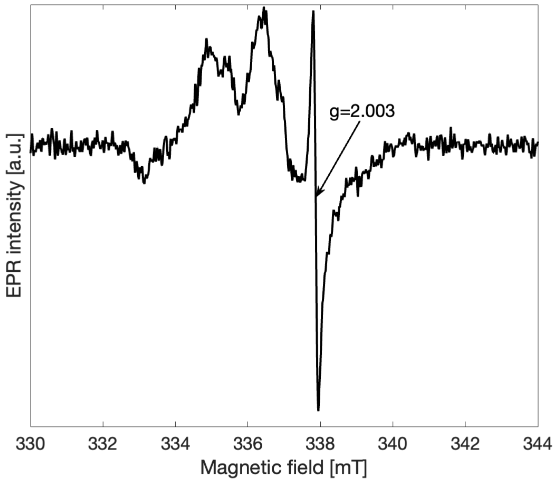

Figure S5: EPR spectrum of gCN recorded after adding H_2O_2, 3%, aqueous, to the sample tube and subsequently drying with nitrogen gas.
